# Polydimethylsiloxane/Magnesium Oxide Nanosheet Mixed Matrix Membrane for CO_2_ Separation Application

**DOI:** 10.3390/membranes13030337

**Published:** 2023-03-14

**Authors:** Muhd Izzudin Fikry Zainuddin, Abdul Latif Ahmad, Meor Muhammad Hafiz Shah Buddin

**Affiliations:** 1 School of Chemical Engineering, Universiti Sains Malaysia Engineering Campus, Nibong Tebal 14300, Pulau Pinang, Malaysia; 2 School of Chemical Engineering, College of Engineering, Universiti Teknologi MARA, Shah Alam 40450, Selangor, Malaysia

**Keywords:** gas separation, mixed matrix membrane, magnesium oxide, nanosheet filler, CO_2_ capture

## Abstract

Carbon dioxide (CO_2_) concentration is now 50% higher than in the preindustrial period and efforts to reduce CO_2_ emission through carbon capture and utilization (CCU) are blooming. Membranes are one of the attractive alternatives for such application. In this study, a rubbery polymer polydimethylsiloxane (PDMS) membrane is incorporated with magnesium oxide (MgO) with a hierarchically two-dimensional (2D) nanosheet shape for CO_2_ separation. The average thickness of the synthesized MgO nanosheet in this study is 35.3 ± 1.5 nm. Based on the pure gas separation performance, the optimal loading obtained is at 1 wt.% where there is no observable significant agglomeration. CO_2_ permeability was reduced from 2382 Barrer to 1929 Barrer while CO_2_/N_2_ selectivity increased from only 11.4 to 12.7, and CO_2_/CH_4_ remained relatively constant when the MMM was operated at 2 bar and 25 °C. Sedimentation of the filler was observed when the loading was further increased to 5 wt.%, forming interfacial defects on the bottom side of the membrane and causing increased CO_2_ gas permeability from 1929 Barrer to 2104 Barrer as compared to filler loading at 1 wt.%, whereas the CO_2_/N_2_ ideal selectivity increased from 12.1 to 15.0. Additionally, this study shows that there was no significant impact of pressure on separation performance. There was a linear decline of CO_2_ permeability with increasing upstream pressure while there were no changes to the CO_2_/N_2_ and CO_2_/CH_4_ selectivity.

## 1. Introduction

Sustainable Development Goal 13 (SDG 13) addressed the importance of climate action, which aims to take urgent action in order to combat climate change. Achieving carbon neutrality in order to mitigate climate change has become a crucial objective as carbon dioxide (CO_2_) keeps on increasing with no sign of stopping. Currently, the CO_2_ concentration is 50% higher compared to that in the preindustrial era, and it is still increasing at an alarming rate due to the advancement of the industrial sector [1,2]. Various carbon capture and utilization (CCU) strategies have been developed, such as cryogenic distillation, pressure-swing adsorption, and chemical absorption to minimize CO_2_ emissions into the atmosphere. Even so, these processes have economical drawbacks. For instance, cryogenic distillation needs to operate at extremely low temperature and high pressure; pressure-swing adsorption requires the use of effective adsorbents such as zeolites, which requires adsorbent regeneration; and chemical absorption involves the use of CO_2_-philic chemicals that are normally amine-based, which need to be treated before being discharged. In general, these processes are energy intensive, require large capital investment, have high operating costs, have large carbon footprints, and require complex process [3]. As such, membranes have become an interesting alternative choice for CO_2_ capture due to less energy consumption, simplicity of operation, and smaller carbon footprint [4].

Polymeric membranes can be classified into two main categories namely glassy and rubbery polymers. Glassy polymers have rigid and stiff polymer chains that act as obstacles for gas to pass through, thus exhibiting high gas selectivity with low permeability in addition to higher mechanical strength relative to rubbery polymer. On the other hand, rubbery polymers have a flexible and fluidic polymeric backbone chain, which cause them to have high gas permeance and low selectivity along with relatively lower mechanical strength as compared to glassy polymers [5]. The phase inversion technique is often utilized in the fabrication of polymeric membranes. However, the performance of pristine gas separation membranes is limited by the balance between the dissolution–diffusion process, which gives rise to Robeson’s upper bound [6,7]. Although, some polymeric material, such as tricyptene-based polymer of intrinsic microporosity (PIM) or thermally-rearranged (TR) polymer, could surpass the upper bound [8,9,10]. Various strategies such as polymer blending, mixed matrix membrane (MMM) fabrication, and thin film composite have been utilized to further maximize the separation performance of pristine polymeric materials [11,12,13]. MMM, especially, received attention due to its simplicity of fabrication and promising improvement in separation performance.

Tantekin-Ersolmaz [14] fabricated PDMS/zeolite 5A with different particle sizes of zeolite 5A. Their result shows that particle size plays a role in improving the permeability of the gas, while the ideal selectivity on the other hand was not greatly influenced. MMM with larger zeolite 5A led to higher permeability improvement compared to MMM with smaller zeolite 5A. This was ascribed to the difficulties of gas permeation due to the enhanced surface area and improved zeolite–polymer interface in the case of smaller zeolite 5A. Madaeni et al. [15] also embedded titanium dioxide (TiO_2_) nanoparticles into a PDMS coating layer supported by porous PES for CO_2_ separation. Their study revealed that there is no major improvement offered by the PDMS/TiO_2_ due to the simultaneous occurrence of copolymerization between Si-OH and PDMS molecules and self-condensation of the polymer chain. As such, the presence of TiO_2_ does not improve the free fractional volume of the PDMS coating to enhance the gas separation performance. Nour et al. [16] blended multiwalled carbon nanotubes (MWCNT) with PDMS for hydrogen purification, and they observed that increasing MWCNT loading from 1 wt% to 10 wt% into PDMS increased H_2_ selectivity due to efficient blocking of CH_4_. Furthermore, they also discovered that the Si-CH_3_ Si-O bond was reduced with the presence of MWCNT, which caused the reduction of CH_4_ permeability. Metal organic framework (MOF) UiO-66 embedded in PDMS at 40 wt% increased CO_2_ permeability and CO_2_/N_2_ selectivity 0.7 times and 1.3 times, respectively, as compared to the base PDMS membrane in a study by Wang et al. [17] Most of the literature describes utilizing PDMS as the membrane and the polymer matrix to form MMM for CO_2_ capture using either fillers with nanorod or nanospherical shaped particles. Recently, two-dimensional (2D) materials have received increasing attention in MMM fabrication due to their potential of exhibiting barrier properties when they are horizontally aligned, thus improving the selectivity of the MMM [18,19]. However, aligning the 2D nanosheet inside the polymer membrane matrix fabricated through typical physical blending still remains a challenge [20].

Zahri et al. [21] incorporated graphene oxide (GO) into a polysulfone hollow fibre membrane, and they reported that the presence of GO inside the polysulfone hollow fibre membrane matrix disrupted the polymer chain packing and increased the tortuosity of the membrane, thus hindering diffusion of gas molecules with larger kinetic diameter, such as nitrogen (N_2_) and methane (CH_4_), while facilitating diffusion of CO_2_ gas through the selective layer, improving CO_2_/N_2_ and CO_2_/CH_4_ gas selectivity. Yang et al. [22] fabricated asymmetric Matrimid polyimide with nitrogen-doped graphene nanosheet MMM with low filler loading (<0.1 wt%). Even with extremely low loading, substantial improvement of gas selectivity was observed; it improved CO_2_/N_2_ selectivity by 45.8% as compared to pristine asymmetric Matrimid membrane with a slight reduction in CO_2_ permeance. The observation was attributed to the increased tortuous path induced by the presence nitrogen-doped graphene in the dense selective layer of Matrimid membrane. Similar observations were also made by Feijani et al. [23]. Furthermore, Asim et al. [24] incorporated a range of 1 wt.% to 10 wt.% of 2D graphitic-Polytriaminopyrimidine (g-PTAP) into Pebax-1657 membrane. The optimum 2D g-PTAP loading they obtained based on the gas separation performance was at 2.5 wt.%, whereby they observed a simultaneous improvement of CO_2_ permeability from 82.3 to 154.6 barrier with CO_2_/N_2_ selectivity improved from 549.5 to 83.5. To conclude, the inclusion of inorganic filler inside a glassy polymeric membrane led to the disruption of the polymeric chain, which resulted in an increased tortuosity path of the gas molecule traveling between the retentate and permeate sides, thus improving the selectivity of the gas. Other than graphene oxide, other porous 2D-based material from zeolites, metal organic framework (MOF), MXene, covalent organic framework (COF), and layered-double hydroxide were also studied for MMM fabrication in gas separation application [25,26]. Nevertheless, literature regarding PDMS MMM utilizing 2D nanosheet filler for CO_2_ capture is scarce. For instance, there are several studies that already utilized 2D nanosheet fillers to fabricate PDMS MMM, albeit for other separation applications. Gou et al. [27] incorporated MFI zeolite nanosheets into PDMS membranes for butane isomer separation application, in which they discovered that the presence of the open-pore MFI zeolite nanosheet enhanced both n-butane permeability and n-butane/i-butane ideal selectivity. Shen et al. [28] utilized GO in PDMS for a propane recovery application. Jafari et al. utilized reduced graphene oxide (rGO) functionalized with octadecylamine for toluene removal using pervaporation [29]. Based on our literature study, we believe that PDMS MMM utilizing 2D nanosheet metal oxide filler is yet to be reported exclusively for CO_2_/N_2_ and CO_2_/CH_4_ separation application.

Magnesium oxide (MgO) has been studied and used as adsorbent, as it has been shown to have affinity towards CO_2_ gases, which can be adsorbed onto the surface of the MgO [30,31,32]. Previous work utilizing MgO nanoparticles as a dispersed phase to form MMM using glassy polymer showed improved gas permeability with slightly reduced gas selectivity, which could be ascribed to the increased formation of free volume in the polymer [33,34]. The focus of this work is to determine the effect of MgO exhibiting hierarchically porous 2D nanosheets with a PDMS matrix. According to Li et al. [35], such 2D porous nanosheet configurations having high surface area, open pore structure, and well-dispersed fine MgO nanoparticles are favourable for CO_2_ gas sorption. Additionally, MgO could potentially facilitate CO_2_ transport through the membrane via Lewis acid–base mechanism, as explained in previous literature [36,37,38], which may contribute to the improvement of the gas separation performance. Secondly, as far as the fabrication of ultra-thin defect-free asymmetric membrane is concerned, they are really challenging, as they have many factors to be considered [39]. Typically, an asymmetric membrane with a thin skin layer experiences incomplete polymer coalescence with pinhole formation, thus reducing the gas selectivity [40], and these defects are commonly sealed with highly permeable rubbery polydimethylsiloxane (PDMS) [41]. Rubbery polymer transports the gas penetrant using gas solubility rather than gas diffusivity through the polymer, as in glassy polymer, due to the flexible polymeric chain backbone of the rubbery polymer [42]. Hence, rubbery polymer exhibits much higher permeability towards the gas penetrant as compared to glassy polymer. However, the balance of solution–dissolution process causes rubbery polymer to show relatively lower selectivity as compared to glassy polymer, which causes it to perform more poorly than the upper bound [7]. PDMS is a rubbery polymer with high permeability towards CO_2_ gas and with considerably good ideal selectivity against CO_2_/N_2_ and CO_2_/CH_4_. It is also cheap and readily available in the market [43,44]. It was hypothesized that inorganic filler with nanosheet structure gives rise to barrier properties, which can enhance gas separation properties [45]. As such, it is expected that the inclusion of 2D nanosheets into rubbery polymers can improve the selectivity of the membrane while achieving high permeability of the gas penetrant. Moreover, the fabrication of asymmetric MMM with inorganic fillers also gives rise to wasteful distribution of the filler in the porous substructure, which does not contribute to the separation process, since the process is dominated by the dense skin layer. Hence, mixed matrix thin film composite (MMTFC) membranes with secondary selective layers made from other highly permeable polymeric materials embedded with inorganic filler appear to be more viable [46]. However, Henis and Tripodi [47] proved that the coating layer does not play a significant role in separation of gas; the separation performance is ultimately dictated by the integral skin layer of the asymmetric membrane. Henceforth, in this study, we focus on determining the effectiveness of PDMS MMM in TFC by fabricating an MMM consisting of self-standing PDMS and MgO nanosheet to elucidate the intrinsic separation performance of PDMS MMM for CO_2_ capture application in flue gas or natural gas separation to determine whether the PDMS MMM coating plays a role in the MMTFC configuration membrane. Moreover, to the best of our knowledge, no literature has reported the use of PDMS with an MgO nanosheet for CO_2_/N_2_ and CO_2_/CH_4_ separation application. PDMS is chosen as the membrane matrix material in this study for its readiness in forming dense membrane and as it is a highly permeable polymer towards CO_2_.

## 2. Materials and Methods

### 2.1. Materials

PDMSs (Sylgard-184) were procured from Dow Corning (USA). They consist of two components: elastomer and curing agent. In accordance with the recommendation by the manufacturer, the elastomer and curing agent were mixed at the ratio of 10:1 for membrane fabrication. The n-pentane (>96 wt% purity) was purchased from Merck (Germany) was used as the solvent for membrane fabrication. Sodium hydrogen carbonate, NaHCO_3_, and magnesium chloride, MgCl_2_ (anhydrous) precursors, were purchased from Merck (Germany) for the fabrication of magnesium oxide nanosheets. Ethanol, C_2_H_5_OH (99.5 wt% purity), was purchased from Merck (Germany). All materials procured were used as received without further purification.

### 2.2. Membrane Fabrication

Dense PDMS membranes were fabricated with a solvent evaporation method [48]. PDMS elastomers were dissolved with a magnetic stirrer for 15 min in n-pentane to form 10 wt% PDMS solution at room temperature. After the solution became homogenous, the curing agent was added and further stirred for 1 h at room temperature to ensure complete dissolution of solution. A fixed volume of the homogenous solution was poured into a levelled Petri dish with a fixed size and heated at 35 °C for 2 h to allow complete solvent evaporation. The rate of solvent evaporation was kept slow to prevent formation of pores or pinholes. The formed membranes were then placed in a vacuum oven at 120 °C overnight before use.

### 2.3. Synthesis of Magnesium Oxide Nanosheet

A facile nonhydrothermal method was utilized to fabricate the MgO nanosheets that were previously studied for solar cell application [49] and olefin/paraffin separation [50]. Briefly, 0.32 g of NaHCO_3_ were dissolved with a magnetic stirrer in a mixture of 100 mL of ethanol and 45 mL of ultrapure water (18.2 MΩ-cm). After all the solids were completely dissolved, 0.1 g of MgCl_2_ were added to the solution and were left for about 3 h to allow complete reaction. The solution was then purged with N_2_ gas at 1 atm and heated to 70 °C for two hours with a plate heater. A white solid formed, and magnesium carbonate (MgCO_3_) was retrieved and centrifuged several times (with 2:1 ratio of ethanol/ultrapure water) to remove residual unreacted reactant. The retrieved white solids were vacuum filtered on filter paper and dried at room temperature before being calcined at 400 °C for 2 h to decompose the MgCO_3_ into MgO. The furnace was ramped at 5 °C min^−1^. The surface area of the synthesized MgO nanosheet particles was characterized by Brunauer–Emmett–Teller (BET) method using Micromeritics ASAP 2020 (Micromeritics Instrument Corp., Georgia, USA). The weighed samples (~0.10 g ± 0.01 g) were vacuumed and heated to 300 °C for 16 h to completely remove any adsorbed moisture on the surface of the synthesized MgO nanosheets.

### 2.4. Mixed Matrix Membrane Fabrication

**Figure 1:**
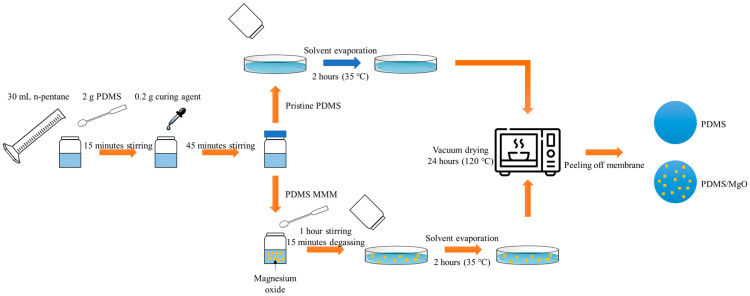
Schematic representation of membrane preparation.

The fabrication of the mixed matrix membrane was similar to the steps reported in Section 2.2. After 1 h stirring at room temperature, and with the addition of curing agent, precalculated MgO nanosheets with various loadings (0, 0.25, 0.5, 1.0, 2.5, 5.0 wt%) with PDMS mass basis were added to the solution under stirring conditions using Equation (1). The mixture was then stirred for another hour and sonicated for 15 min to ensure good dispersion of the filler inside the solution. A fixed volume of the solution was then poured to a fixed-size Petri dish and heated at 35 °C to allow complete evaporation of the solvent. The membranes were then heated at 120 °C for 24 h before being tested using a gas separation test. Preparations of neat and MMM PDMSs are shown in Figure 1. The fabricated membranes were denoted M1 to M6, where the mass component of each MMM is shown in Table 1.

(1) $$Filler\left(\%\right)=\frac{Mass\;of\;MgO\left(g\right)}{Mass\;of\;PDMS\left(g\right)+Mass\;of\;MgO\left(g\right)}$$

### 2.5. Membrane Characterization

The surface and cross-sectional area of the fabricated membranes were observed using tabletop scanning electron microscopy (SEM) (TM3000, Hitachi Ltd., Tokyo, Japan) to study the distribution of the fillers. A water contact angle (CA) measurement was made using a goniometer (Rame’-Hart Instrument Co., Succasunna, NJ, USA), with a sessile drop method to study the surface properties of the membranes. A constant volume of deionized (DI) water was dropped on the membranes at five different locations at room temperature to obtain the average values. Average values of five readings are reported for the CA measurement. Fourier transform infrared (FTIR-ATR) (Perkin Elmer, Waltham, MA, USA) was used to study the bonds present inside the membranes and to confirm the presence of magnesium oxide nanosheet fillers. Brunauer–Emmett–Teller (BET) analysis was performed on the fabricated MgO nanosheets to determine the isotherm and surface area. Thermal analysis of the fabricated membranes was performed using thermal gravimetric analysis (TGA) (STA6000, Perkin Elmer, USA) and differential scanning calorimetry (DSC) (DSC4000, Perkin Elmer, USA) to determine the change in the physical or thermal transition of the fabricated membranes to further elucidate the interaction between PDMS and MgO. For TGA, the samples were heated at a rate of 10 °C/min from 25 °C to 800 °C under inert nitrogen atmosphere condition while for DSC analysis, the samples were heated at a rate of 10 °C/min from 25 °C to 400 °C.

### 2.6. Gas Separation Performance

The gas performance set-up and its schematic diagram has been described elsewhere [44]. Pure gas separation performance was conducted at pressure ranging from 2 to 5 bar at room temperature conditions using a constant-volume variable pressure method. All the gas used was of high purity (>99.995 wt%). The flat sheet PDMS membrane samples were fixed to a module with an effective permeation area of 3.142 cm^2^. The pure gases were fed in the following sequence: N_2_, followed by CH_4_, and lastly CO_2_. The upstream was purged with targeted test gas before commencing the test, and all the data were taken at least twice to ensure consistency. The permeability of the individual gas was calculated using Equation (2):

(2) $$P=\frac{273.15\times 10^{10}\times V\times l}{\frac{760AT\left(P_{o}\times 76\right)}{14.696}}\left(\frac{dp}{dt}\right)$$

where *P* is the gas permeability in unit Barrer (1 Barrer = 1 × 10^−10^ cm^3^(STP)∙cm/cm^2^·s·cmHg), *V* is the volume of gas permeate in the downstream chamber (12 cm^3^), *l* is thickness of the effective skin layer (cm), *A* is the effective permeation area (cm^2^), *P_o_* is the upstream pressure (psi), and *dp/dt* is the pressure gradient (psia/s). The gas ideal selectivity is then calculated using Equation (3):

(3) $$\alpha_{i,j}=\frac{P_{i}}{P_{j}}$$

where *α* is the ideal gas selectivity (ratio of individual gas permeability), and *P* is the permeability of the individual gas calculated using Equation (2). The subscripts *I* and *j* correspond to the gas penetrant species.

## 3. Results and Discussions

### 3.1. Characterization of MgO Nanosheet Filler

**Figure 2:**
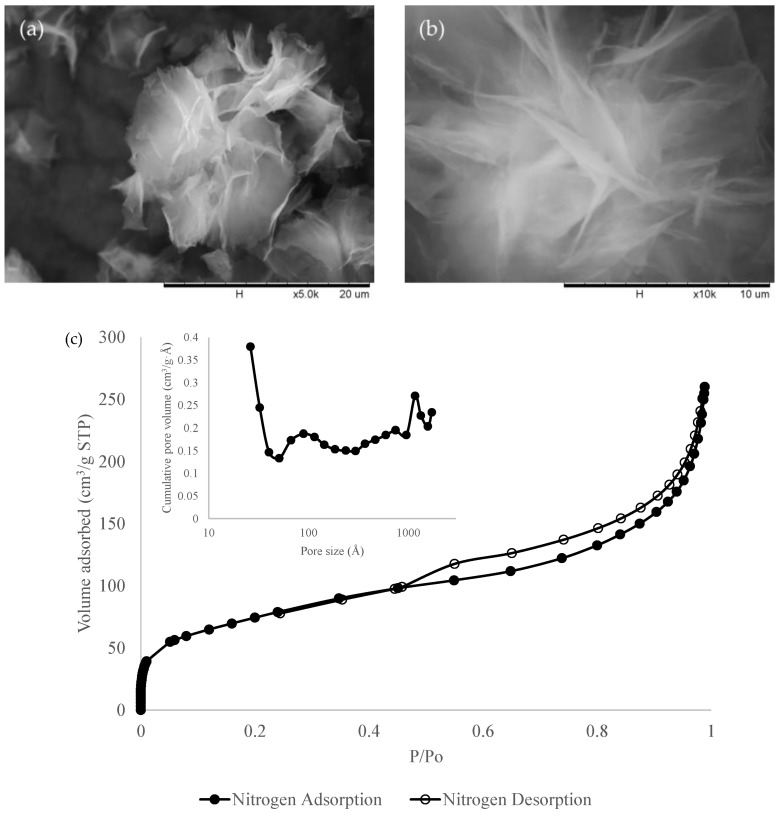
SEM image of MgO nanosheet at (**a**) 5000× magnification and (**b**) 10,000× magnification. (**c**) N2 adsorption–desorption isotherm of synthesized MgO nanosheet. The inset shows the BJH pore distribution curve of the MgO nanosheet.

The as-synthesized mesoporous MgO nanosheets were characterized using SEM imaging and N_2_ adsorption isotherm using the BET method presented in Figure 2. From the SEM images, the as-synthesized MgO nanoparticle exhibited a hierarchically nanosheet-like structure. It was further analysed with N_2_ adsorption–desorption isotherm, where the N_2_ adsorption isotherm exhibited a type IV isotherm with a hysteresis loop characteristic showing the synthesized MgO nanosheet exhibiting multi-modal pores [50]. The isotherm also showed a steep rise at P/P_0_ = 1, indicating the presence of pores due to the voidage between the aggregated MgO nanosheet particles [51]. The BET surface area of the synthesized MgO nanosheets obtained in this study is 270 m^2^/g, slightly higher than the BET value reported by Qureshi et al. [49], which is at 216 m^2^/g. The pore size distribution of MgO nanosheets was determined using the Barrett–Joyner–Halender (BJH) curve as shown in the inset of Figure 2. The synthesized MgO nanosheet exhibited four different maximum points of pore distribution at 2.6 nm, 8.9 nm, 74.7 nm, and 117.1 nm, with broad pore distribution between 30 and 95 nm based on the BJH pore distribution curve indicating a hierarchically arranged three-dimensional structure. These values were close to those from a previous study by Park et al. [50] who used the MgO nanosheet for an olefin/paraffin separation process, although it is slightly differs from Qureshi et al. [49], who achieved only a single maximum peak in the range of approximately 10–15 nm. The average thickness of the synthesized MgO nanosheet was estimated based on FESEM. The average thickness was taken from four different places. A sample image of the thickness measurement is shown in Figure S1(see Supplementary Materials).

### 3.2. Morphology of PDMS and PDMS/MgO Membrane

**Figure 3:**
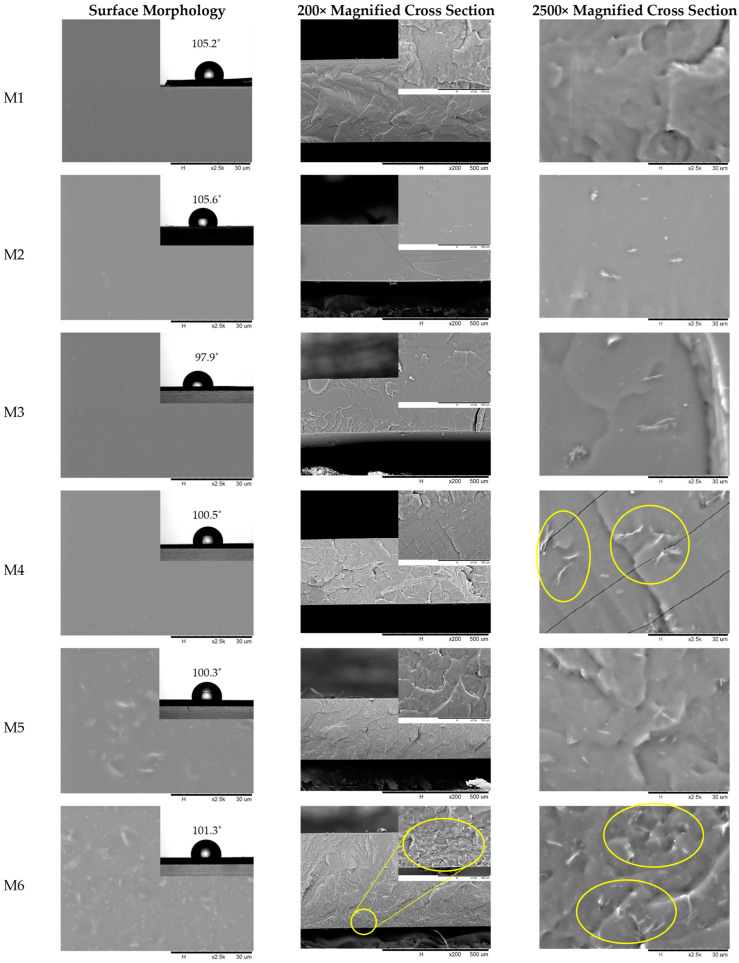
Surface and cross-sectional morphology of PDMS and PDMS/MgO membranes at various loading. The inset of the surface morphology shows the water CA obtained from five average values. The inset in cross-sectional area of M6 shows the sedimentation of MgO nanosheet at the bottom of the membrane matrix.

The cross-sectional and surface morphology of the fabricated membranes were examined using SEM images, as are shown in Figure 3, to study the effect of the presence of MgO nanosheets on membrane structure. The surface of the M1 to M6 membranes appeared dense with no sign of pinhole formations. When lower loading of MgO was embedded into the PDMS matrix, some of the MgO was observed on the surface of the membrane (M2, M3, and M4). Meanwhile, when the loading was higher, more of the MgO particle appeared on the top surface (M5 and M6). To see whether this phenomenon affected the surface properties of the membrane, water CA of the membranes were measured. For a pristine M1 membrane, the water CA obtained was 105.2°, which falls in the hydrophobic category. The value obtained was also close to that of other reported literature [52,53]. MgO is known to be hydrophilic [54]. The presence of MgO filler in MMM slightly altered the CA of the MMM, as some part of this filler appeared on the surface of the MMM. The CA of M2 was essentially similar to M1, as the MgO loading was very low (0.25 wt%). However, the CA started to reduce when the loading was further increased. Regardless, the difference of the CA values was not significant, even though it appeared to drop with increasing MgO loading. Based on the observation of the cross-sectional morphology, all membranes exhibited a dense layer across the cross section of the membrane. The rate of liquid–liquid demixing from the polymer–solvent–nonsolvent system determined the structure of the fabricated membrane. Slow liquid–liquid demixing will lead to a dense or spongy structure while fast liquid–liquid demixing will lead to an elongated, finger-like structure. In this study, all membranes were fabricated using the solvent evaporation method. The solvent used to dissolve the PDMS precursor in this study, n-pentane, is a volatile solvent. When a dope solution is poured into a Petri dish of a fixed size, n-pentane will gradually evaporate from the polymer–solvent system, thus leaving only PDMS in the petri dish. The PDMS polymer solution also gradually becomes more concentrated until all n-pentane eventually evaporates out. Hence, the PDMS polymer has sufficient time to rearrange itself neatly, forming a dense PDMS membrane. The thickness of the fabricated membranes is listed in Table S1. The thickness of M1 and M6 varied slightly compared to the other membranes, which could be due to the error introduced when levelling the Petri dish during solvent evaporation. However, it is important to note that the thickness of the membrane may affect the gas permeance, but it does not affect the permeability values of the membrane, as permeability is an intrinsic property of the membrane. However, in the case of a thin membrane (<200 nm), it is reported that permeability and selectivity of the PDMS membrane become thickness-dependent [55,56]. Moreover, the thickness of the membranes fabricated in this study were greater than 250 μm. Hence, it is expected that a slightly varied membrane thickness will not affect permeability and selectivity of the membranes in this study. Furthermore, the surface of neat PDMS (M1) appeared smooth and defect-free, without formation of pinholes, showing that dense PDMS membrane was successfully fabricated with 10 wt.% PDMS solution in n-pentane with the solvent evaporation method.

For MMM fabrication, there are several important factors that must be considered, such as the compatibility between polymer and filler, their interaction, and the dispersion of the filler inside the polymeric membrane matrix. The inorganic fillers need to be evenly distributed throughout the polymeric matrix to ensure that there is no local agglomeration inside the membrane, which can create voids and form nonselective channels, which is not favourable for gas separation as it will affect gas selectivity. Based on the cross-sectional image of M2 and M3, there was no sign of aggregation of the filler, and the filler appears to be well dispersed. The surfaces of the M2 and M3 membranes also appeared smooth, which means that the MgO nanosheet did not migrate and protrude to the surface, forming nonselective voids. However, in M4 with 1 wt.% of MgO loading in PDMS, there appeared to be some aggregate of the inorganic filler inside the polymer matrix (circled in yellow), although the agglomeration was not really severe to the point where they stacked with each other in a manner that could form a visible nonselective void. The phenomena also occurred in M5, where there were more agglomerates of MgO nanosheets observed. The agglomeration of nanoparticles often happened at high loading, as they possess high surface energy. The interaction between filler due to Van der Waals or other interaction [57,58] became more dominant than the interaction between the filler and polymer matrix at high filler loading, thus forming an agglomerate of fillers, which may lead to the formation of a nonselective void.

However, M6 does not only have an agglomerated filler, but the membrane also exhibits significant sedimentation of MgO nanosheet filler on the bottom side of the membrane, as shown in Figure 3 (inset cross-sectional image of M6). The excessive loading of the MgO nanosheet filler inside the polymer matrix may have restricted the homogeneity of the dope solution, thus preventing the filler from being well dispersed inside the polymer matrix. The same phenomenon can be observed in another study by Jusoh et al. [59], where it was observed that the zeolite T filler started to form sedimentation at the bottom of the membrane at higher zeolite T loading. Inadequate strong adhesion between the MgO nanosheet filler and the PDMS polymer chain may also have contributed to the sedimentation of the filler as well. According to Chang Y.-W. and Chang B.K. [60], another aspect to be considered for the sedimentation phenomena is the viscosity of the polymer dope. Sedimentation easily occurs with lower polymer dope viscosity as it is not enough to keep the filler suspended during phase separation. On the other hand, it is harder for filler inside a more viscous polymer dope to experience sedimentation due to competing forces between gravitational pull and hindrance from the viscosity of the polymer dope. In the context of this study, the PDMS concentration was maintained at 10 wt.% for all fabricated membranes. As such, at lower filler concentration, the viscosity of the polymer dope solution was sufficient to ensure that the filler be suspended during phase separation. While at higher filler concentration, the influence of viscosity becomes less significant, and the effect of gravitational force acting on the filler becomes dominant, thus leading to sedimentation. As such, it can be concluded that the combined effect of the weak adhesion between MgO the nanosheet filler and the PDMS polymer chain and the dominating effect of gravitational pull on the filler at higher loading leads to sedimentation of the filler. The opposing nature of hydrophobic solvent (n-pentane) and hydrophilic fillers (MgO) may or may not contribute to the sedimentation of the fillers. The sedimentation issue may be mitigated by increasing the polymer dope solution, which in turn increases the viscosity of the polymer solution, as it is known that viscosity of a polymer is a function of its concentration [44,61,62]; hence, the higher polymer viscosity may keep higher filler loading suspended during phase separation. However, this will also lead to thicker membrane formation, which in turn will lower the gas permeance due to the increased resistance from the membrane’s thickness. Therefore, in this study we did not further increase the polymer concentration. Hence, it is concluded that the MgO nanosheet can be dispersed effectively in a PDMS membrane matrix up to 1 wt.% content.

### 3.3. FTIR Analysis

**Figure 4:**
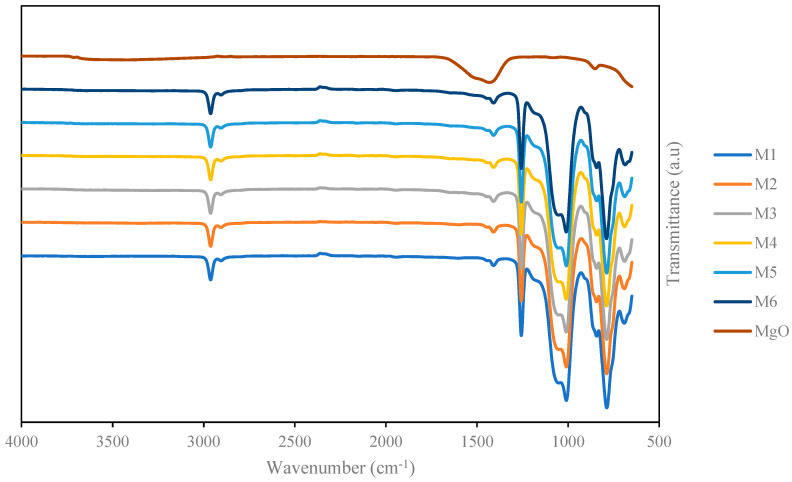
FTIR spectra analysis of PDMS and PDMS/MgO membrane.

The full FTIR spectra is shown in Figure 4, while Figure S2a shows the magnified spectra from 600 to 1700 cm^−1^, and Figure S2b shows the difference in the intensity of the peak in the region from 600 to 1700 cm^−1^. For pristine PDMS, the peak at 787 cm^−1^ corresponds to the stretching of the -CH_3_ and Si-C bond in the Si-CH_3_ chain. The peak from 1009 to 1061 to cm^−1^ shows the Si-O-Si stretching of the polymeric chain. The peak at 1255 cm^−1^ is assigned to the deformation of the -CH_3_ group in the Si-CH_3_ chain, and, finally, the stretching at 2957 cm^−1^ is due to the asymmetric stretching of -CH_3_ in the polymeric chain of the PDMS polymer [63]. A freshly prepared MgO nanosheet particle was used for the FTIR spectra analysis. The broad peak ranging from 600 to about 800 cm^−1^ indicates the Mg-O-Mg bond [64], and the characteristic peak at 850 cm^−1^ is ascribed to the stretching of the Mg-O metallic bond of the MgO nanosheet particle [65,66]. Meanwhile the broad peak at 1417 cm^−1^ is assigned to the stretching of symmetrical and asymmetrical carboxylate O-C=O, which may present due to the unreacted precursors [67]. There exists a very minimal peak in the range from 3000 to 3800 cm^−1^ that is attributed to the presence of the H-O-H bond of the water molecule on the surface of the MgO nanosheet nanoparticle [68]. It is known that MgO is highly hygroscopic; thus, the moisture from the atmosphere may readily adsorb onto the surface of the MgO nanoparticle [31,69]. Additionally, no further peak emerged from the spectra analysis in MMM, showing that there was no chemical interaction between PDMS and MgO nanosheets other than the slight differences in the intensity of the peak at 850 cm^−1^ and 1417 cm^−1^, which corresponds to the amount of MgO present in the fabricated MMM. The FTIR spectra also confirmed that there is no presence of residual n-pentane solvent in the fabricated membrane using the solvent evaporation method.

### 3.4. Thermal Gravimetric Analysis (TGA) and Differential Scanning Calorimetry (DSC)

**Figure 5:**
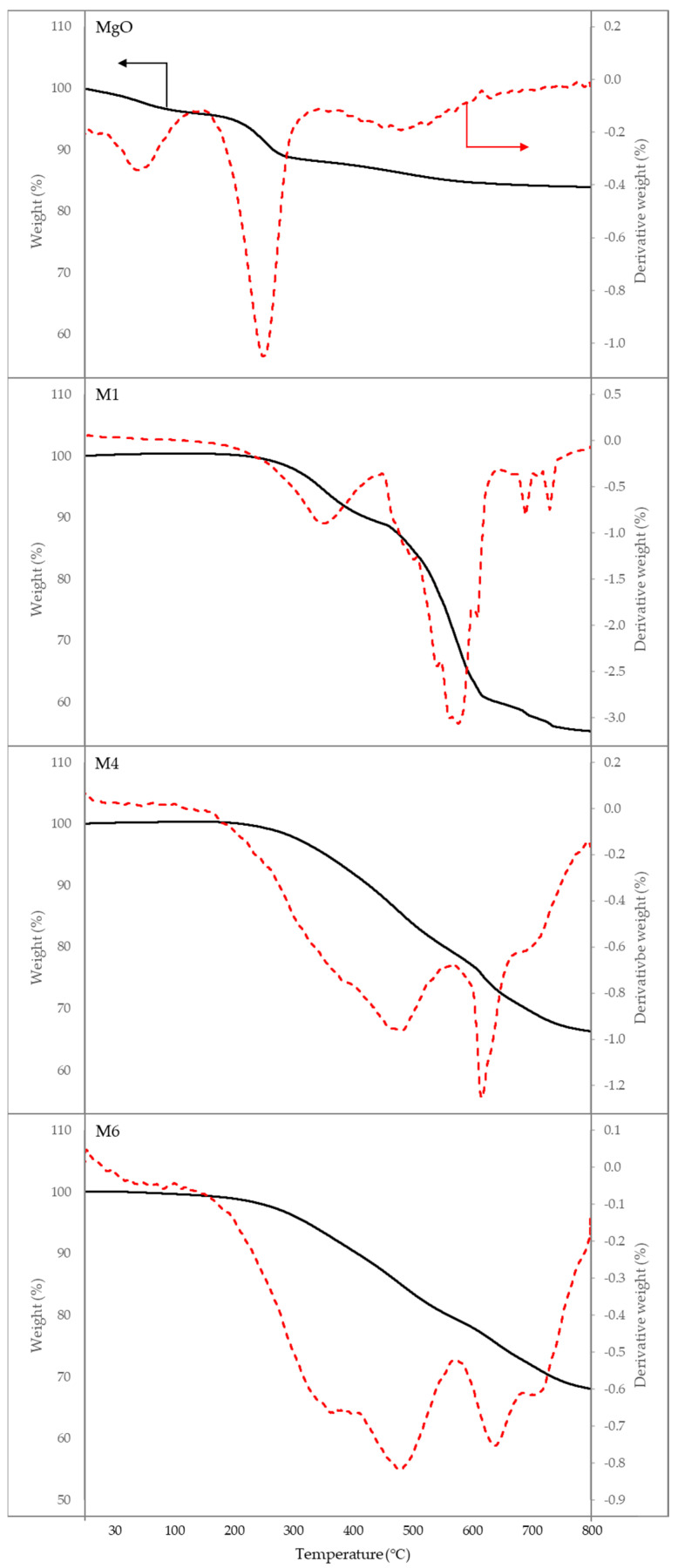
Thermal gravimetric analysis (TGA) and derivative thermogravimetric (DTG) curves of PDMS and PDMS/MgO membranes.

The thermal stability of PDMS and PDMS/MgO membranes was studied by analysing the thermal decomposition of the membrane with TGA and DTG curves, as shown in Figure 5. For MgO nanosheet powder, the temperature loss at the 200 °C to 300 °C region could be ascribed to the loss of the water moisture that may have physically or chemically adsorbed on the surface of the MgO nanosheet powder upon long storage [69,70]. For pristine PDMS membranes, the first on-set temperature ranged from approximately 300 °C to 400 °C, which is due to the typical loss of water moisture and physical adsorbed layer [71] as well as removal of lateral groups from the main chain of PDMS, while the mass reduction from around 450 to 600 is ascribed to the breakdown of the PDMS chain [48]. The weight loss for M4 and M6 was less than that of M1 based on the TGA and DTG curves in Figure 5. The final residues remaining on M1, M4, and M6 were 55%, 66%, and 68%, respectively. This could be ascribed to the amount of MgO nanosheet filler present in each membrane, whereby M6 had the highest MgO nanosheet loading, contributing to more residual mass compared to M4 and M1. The initial thermal decomposition of PDMS/MgO MMM 393 shifted to 501 °C for both M4 and M6 as compared to the pristine M1 membrane, which could be ascribed to the disruption of the polymeric matrix by the MgO nanosheet lowering the polymer chain’s mobility [72]. The thermal stability between M4 and M6 does not differ significantly, albeit the MgO nanosheet content in M6 is about five times higher than in M4. Both M4 and M6 exhibited almost similar TGA curves. However, based on the DTG curves, M4 showed a slightly higher rate of mass loss at 509 °C (−0.964%) and 635 °C (−1.25%) as compared to the rate of mass loss for M6 at 512 °C (0.818%) and 659 °C (−0.752). Hence, the improvement of thermal stability of the membrane with MgO nanosheet loading beyond 1 wt.% was not significant. Therefore, it can be concluded that the presence of the MgO nanosheet inside the PDMS matrix enhanced the thermal stability of the membrane. The MgO nanosheet prevented volatile movement of volatiles during thermal treatment. When the samples were further heated, M1 showed significant mass loss when the temperature was approximately 598 °C. Meanwhile, for M4 and M6, the second thermal decomposition shifted to 636 °C and 656 °C, respectively. Lastly, the increase in mass residue of the samples could be attributed to the presence of inorganic MgO nanosheet filler that sustains even at high temperature. 

**Figure 6:**
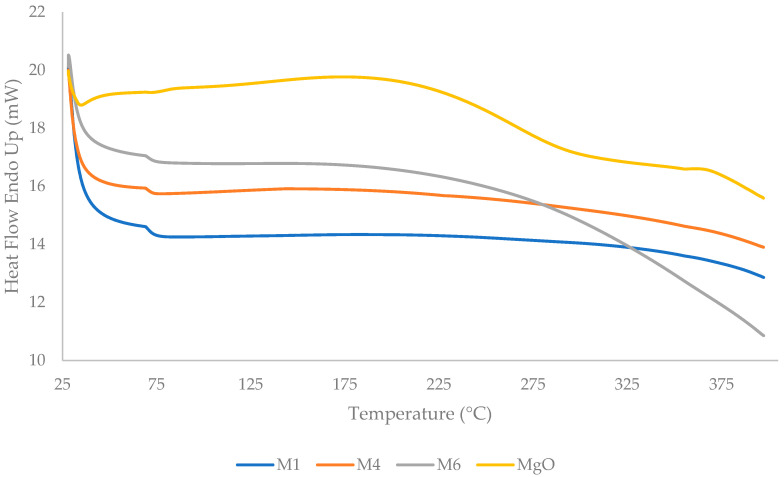
Differential scanning calorimetry (DSC) thermogram of MgO nanosheet powder, PDMS, and PDMS/MgO membranes.

DSC was performed onto the synthesized MgO powder, PDMS, and PDMS/MgO nanosheet composite membrane with temperatures ranging from 25 °C to 400 °C and is represented in Figure 6. For the MgO nanosheet powder, the broad endothermal peak in the range between 175 °C and 300 °C was due to the loss of water moisture [66] as confirmed by the DTG thermogram in Figure 5. Meanwhile, the first thermal transition was observed at the region between 60 °C and 75 °C for M1, M4, and M6. The transitional temperature did not differ much between M1, M4, and M6, but the heat flow value increased with increasing MgO nanosheet content in the PDMS membrane. Suleman et al. [73] also reported nearly similar first-transition temperatures in their studies. Additionally, the endothermic peaks of M1, M4, and M6 in the range between 375 °C and 400 °C were due to the loss of water moisture and physical adsorbed layer from the membranes, as discussed in Figure 5. There is no distinctive glass transition temperature (T_g_) observed in Figure 6. Moreover, PDMS is one of the rubbery polymers that has the lowest T_g_ (~−123 °C) [74].

### 3.5. Gas Separation Performance

#### 3.5.1. Effect of MgO Nanosheet Loading

**Figure 7:**
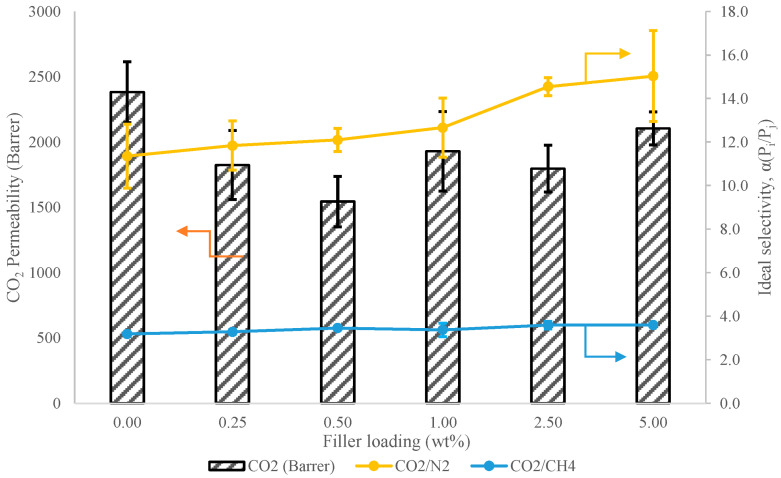
Gas separation performance of PDMS and PDMS/MgO with respect to various filler loading at ∆P = 2 bar and T = 25 °C.

Single gas permeation tests are carried out on the fabricated PDMS and PDMS/MgO membranes to further elucidate the effectiveness of MgO nanosheets as fillers in PDMS membranes. In this section, the upstream pressure was kept at 2 bar, and the permeation was carried out at room temperature. Figure 7 shows the CO_2_ permeability with CO_2_/N_2_ and CO_2_/CH_4_ as ideal selectivity of the membranes with respect to various MgO nanosheet loadings. Based on the trend, the CO_2_ permeability did not show a linear trend with the increase in MgO nanosheet filler loading. The pristine PDMS membrane exhibited 2382 Barrer with CO_2_/N_2_ and CO_2_/CH_4_ ideal selectivity of 11.8 and 3.2, respectively. The CO_2_ permeability value of pristine PDMS reported in this study was slightly lower, while the CO_2_/N_2_ selectivity was slightly higher as compared to other literature that used n-hexane to fabricate the PDMS membrane. In this study, n-pentane was used to fabricate the PDMS membranes, compared to other studies that used n-hexane [75]. The use of different solvents may have contributed to this observation. Results regarding this phenomenon have been demonstrated in several studies that show the use of different types of solvents will lead to varied permeability and selectivity values of the membranes due to the differences of boiling point of the solvents and solubility parameters between the solvents used and the polymers [75,76,77]. Furthermore, when MgO nanosheet filler was introduced into the PDMS matrix at 0.25 and 0.5 wt.%, the CO_2_ permeability showed a declining trend accompanied with minor improvement over CO_2_/N_2_ and CO_2_/CH_4_ ideal selectivity. The CO_2_/N_2_ and CO_2_/CH_4_ ideal selectivity increased from 11.8 to 13.5 and from 3.2 to 3.5, respectively, when the PDMS membrane was loaded with 0.5 wt.% filler as compared to pristine PDMS membrane. However, upon incorporation of MgO nanosheets up to 0.5 wt.%, the CO_2_ permeability was reduced. Such phenomena are common in the fabrication of MMM, where the presence of filler disrupts the polymeric chain arrangement [78]. Typically, for glassy polymer, the presence of inorganic filler often causes disruption of the polymer chain that leads to the increment of free fractional volume of the glassy membrane. The case can be confirmed with increased gas permeability and selectivity in gas separation performance of MMM as compared to a pristine glassy membrane [79,80]. However, PDMS is a rubbery polymer with a flexible and highly mobile polymeric chain rather than a strong and rigid polymeric chain, as possessed by glassy polymers. Similarly, the presence of MgO nanosheets disrupts the polymeric chain of the PDMS membrane matrix. Although, rather than increasing the free fractional volume, it impedes the mobility of the polymer chain of PDMS membranes, thus reducing the flexibility of the polymer chain with the presence of an MgO nanosheet inside the polymer matrix, hence, reducing the permeability of the gas, as it is difficult for the gas penetrant to diffuse and move within the polymeric chain. Madaeni et al. [15] suggested two mechanisms to explain the phenomena, namely the copolymerization between Si-OH groups and PDMS molecules and the self-condensation of polymer chains. Moreover, the enhancement of hydrophilicity of PDMS is not favourable. As a result, the presence of hydrophilic MgO nanosheets does not improve the free volume fraction of PDMS membranes. In tandem with the reduced gas permeability, CO_2_/N_2_ and CO_2_/CH_4_ ideal selectivities were observed to increase slightly, as stated previously. It has been long postulated that nanosheet fillers with high aspect ratios of length to thickness oriented perpendicularly to the gas diffusional path exhibit barrier properties that could enhance gas separation performance as discussed by DeRocher et al. [81] and Kang et al. [82]. Although in this study, the orientation of the nanosheet was not expected to be perpendicular to the gas diffusional path as the membrane was fabricated through the typical solvent evaporation method. The orientation was most likely randomized [20]. The high aspect ratio of the nanosheet filler increased the tortuosity of the diffusional path for the gas penetrant to pass through the dense selective layer of the membranes. Hence, the gas penetrant needed to cover more distance of diffusional path before it reached the permeate side, which led to the reduction of gas permeability. Nevertheless, the formation of these paths allowed a gas with smaller kinetic diameter, CO_2_ (d = 3.3 Å), to diffuse more easily as compared to gases with larger kinetic diameters, such as N_2_ (d = 3.64 Å) and CH_4_ (d = 3.8 Å). This explained the reduction of CO_2_ permeability with slight improvement towards the ideal gas selectivity similarly observed in other literature [21]. Another factor that could possibly contribute to the reduced gas permeability with improved selectivity could be the rigidification of the polymer chain at the polymer filler interface. As observed in a study by Ehsani and Pakizeh [83], the trend of reduced permeability with increased gas selectivity could either be ascribed to the rigidification of the polymer–filler interface or due to pore blockage of the filler by the polymer. However, in this study, the reduction of gas permeability was more likely influenced by the rigidification in the polymer–filler interface region, as the MgO nanosheet is not a filler with porous channels. Thus, it is not possible for pore blockage to occur. Similar trends can also be observed in a study by Tao Li et al. [84]. Due to rigidification, the polymer–filler contact is stronger, thus restricting the polymer chain mobility, which promotes enhancement of CO_2_/N_2_ and CO_2_/CH_4_ ideal selectivity [85].

However, the CO_2_ permeability increased from 1544 Barrer to 1927 Barrer when the filler loading was further increased from 0.5 wt.% to 1.0 wt.%. This could possibly be due to the small aggregation formed on some local part of the PDMS matrix leading to a defective area in the MMM, as shown in the inset image of the SEM in Figure 3. The small aggregation formed may have caused the formation of small interfacial void in the polymer–filler interface causing the gas molecules to move through these voids, thus contributing to increased gas permeability. Additionally, the ideal gas selectivity did not show a declining trend, as the aggregate was not so severe that it caused the formation of nonselective voids as observed in the image. Thus, it can be said that the voids formed at the interfacial contact at the polymer–filler interface were not major and were capable of discriminating the gas through molecular sieving [86,87].

Lastly, when the filler was further increased to 5 wt.%, the gas permeability increased to 2104 Barrer. The gas separation performance concurred with the inset of the cross-sectional SEM image of M6 in Figure 3, where there is sedimentation of the MgO nanosheet at the bottom of the MMM following its complete phase separation. The deposition of the MgO nanosheet at the bottom of the PDMS membrane matrix may have created nonselective voids in the membrane matrix that promoted the increment of CO_2_ permeability. Regardless, it can be observed from Figure 7 that the CO_2_/N_2_ ideal selectivity does not drop, while CO_2_/CH_4_ ideal selectivity remained relatively constant. Although sedimentation of the fillers was observed, since the fabricated membrane was fully dense, the interfacial voids formed at the sedimented filler section at the bottom of the membrane matrix did not impede ideal selectivity, as there was still a large, dense selective layer with a distributed MgO nanosheet on the top part of the membrane matrix. As such, it is inferred that the filler sediment provided a low resistance diffusional pathway due to interfacial void formation, while the top layer provided a selective diffusional pathway akin to an asymmetric membrane. However, the CO_2_/N_2_ ideal selectivity also showed the highest error bar due to lower reproducibility.

#### 3.5.2. Effect of Variable Pressure

Based on the previous section, the M4 showing the best separation properties with good dispersion of the MgO nanosheets in MMM showing desirable morphology without showing any sign of significant agglomeration was chosen to be studied regarding the effect of feed pressure on the gas separation performance in this section.

**Figure 8:**
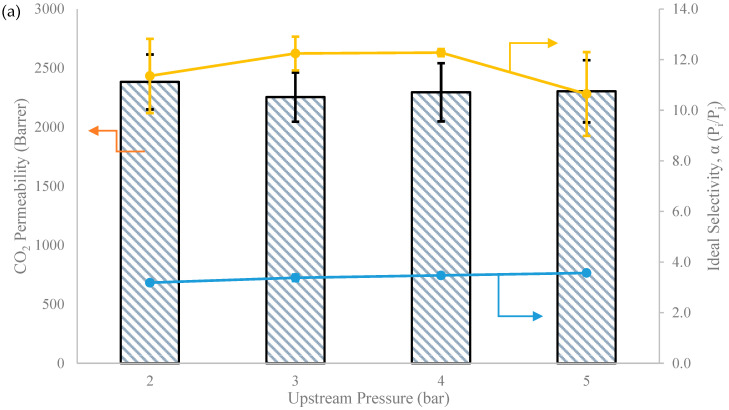

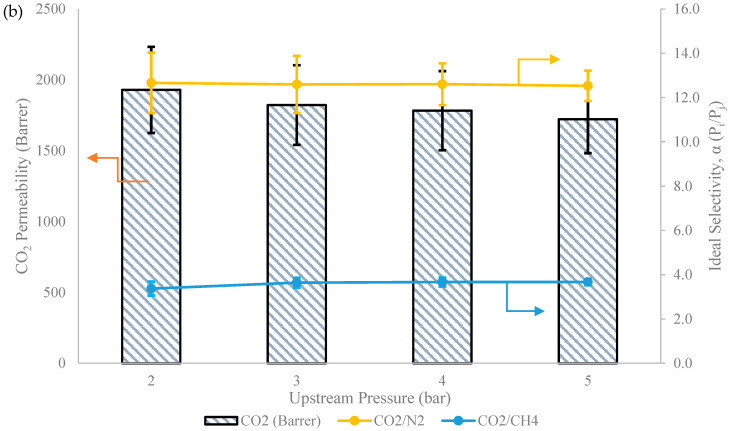
Gas separation performance of (**a**) M1 and (**b**) M4 at various pressures ranging from 2 to 5 bar at room temperature (T = 25 °C).

Based on the trend observed in Figure 8, when the M1 membrane (Figure 8a) was subjected to increased upstream pressure from 2 to 5 bar, the permeability of CO_2_ remained steadily constant at about 2300 Barrer, while the CO_2_/N_2_ ideal selectivity increased slightly from 11.4 to 12.2 and from 2 to 3 bar and dropped from 12.7 to 10.6, with CO_2_/CH_4_ remaining relatively constant with increased upstream pressure. As permeability is an intrinsic property of the membrane, it was expected that it would not change with transmembrane pressure unless the membrane experienced plasticization, where drastic permeability and ideal selectivity reduction could be observed [88]. As for M4 (Figure 8b), which contained 1 wt.% of MgO nanosheet filler, CO_2_ permeability dropped very slightly from 1929 Barrer to 1722 Barrer with no improvement of CO_2_/N_2_ and CO_2_/CH_4_ ideal selectivity as a function of upstream pressure. This behaviour can be explained with the dual-sorption model, where the sorption of gas molecule to the rubbery phase of polymer is governed by Henry’s law, while the adsorption of gas molecule in the microvoid region of the polymer is described by Langmuir’s behaviour [73,89]. At first, CO_2_ solubility increased with pressure in accordance with Henry’s law but soon became obsolete at higher pressure due to shrinkage of the microvoid inside the polymer due to Langmuir’s behaviour [90]. Additionally, it is also possible that the presence of the MgO nanosheet increased the crystallinity of the PDMS MMM at the polymer–filler interface, although it is not quantified in this study. The crystalline region at the polymer–filler interface exhibited reduced gas penetrant solubility and diffusivity as crystallite is impermeable, hence contributing to the reduction of gas permeability [91,92]. Membrane-softening phenomenon also occurred with increased pressure. The trend of our finding regarding the effect of pressure on gas permeability of MMM is also in line with several published studies [93,94]. Table 2 shows the comparison of membrane performances based on PDMS MMM in this study and with other studies.

## 4. Conclusions

PDMS embedded with MgO nanosheets to form MMM was successfully fabricated and tested for CO_2_ separation application. MgO nanosheets were fabricated using a nonhydrothermal method with cheap precursors. The MgO nanosheets showed good dispersion inside the PDMS polymer matrix up to 0.5 wt.%. At 1 wt.%, there was small, local aggregation, although it did not agglomerate and form nonselective voids. Sedimentation of MgO nanosheets was observed at 5 wt.% due to the significant difference of physical properties between the filler and the polymer dope suspension. The incorporation of MgO nanosheets into the PDMS matrix showed improved ideal selectivity as compared to the base PDMS membrane. The presence of MgO nanosheets disrupted the polymer chains and increased the tortuous path of gas penetrant, thus resulting in reduced gas permeability with increased ideal selectivity. PDMS is commonly used to seal the defective surfaces of hollow fibre membranes. As such, we believe that the hybrid PDMS/MgO membrane may be an interesting choice to be used as a coating to further improve the gas separation performance of a mixed matrix thin film composite hollow fibre membrane, although the improvement brought about by the PDMS/MgO layer is marginal. Nevertheless, single gas permeation is not sufficient to justify the performance of the membrane in the real plant condition. Hence, for future work, mixed gas permeation performance would be more useful to determine the effectiveness of MgO nanosheets in improving the separation performance of PDMS membranes.

## Figures and Tables

**Table 1:** Mass composition of the prepared membrane.

Membrane Sample	Elastomer to Curing Agent Ratio	Mass of PDMS (g)	Mass of Curing Agent (g)	Mass of n-Pentane (g)	Mass of MgO Nanosheet (PDMS Mass Basis) (g)
M1	10:1	2	0.2	19.8	0
M2	10:1	2	0.2	19.8	0.00501
M3	10:1	2	0.2	19.8	0.01005
M4	10:1	2	0.2	19.8	0.02020
M5	10:1	2	0.2	19.8	0.05128
M6	10:1	2	0.2	19.8	0.10526

**Table 2:** Gas performance comparison of PDMS MMM with other studies.

Filler	Condition ^1^	CO_2_ Permeability (Barrer)	Gas Pair Selectivity ^2^		Reference
			CO_2_/N_2_	CO_2_/CH_4_	
Mg-MOF-74 (0.46 mmol)	4 bar 30 °C	1608	17.6	-	[38]
Mn-MOF-74 (0.46 mmol)		1466	18	-	
Co-MOF-74 (0.46 mmol)		1508	17.9	-	
Ni-MOF-74 (0.46 mmol)		1502	14.5	-	
MWCNT (1 wt.%)	14 psi	1500	11.83	2.73	[48]
ZSM-5 (66 wt.%)	-	11648	11.1	4.36	[95]
SAPO-34	20 bar	5753	31	4.92	[72]
Zeolite 3A (40 wt.%)	5 bar 35 °C	3125	-	2.95	[96]
Zeolite 4A (40 wt.%)		3208	-	3.09	
Zeolite 5A (40 wt.%)		3137	-	2.97	
MgO nanosheet (1 wt.%)	2 bar	1929	12.7	3.4	This work
	3 bar	1822	12.6	3.6	
	4 bar	1782	12.6	3.7	
	5 bar	1722	12.5	3.6	

^1^ All tests are conducted at 25 °C unless stated otherwise. ^2^ The reported gas selectivity is for single gas permeation (ideal selectivity).

## Data Availability

Not applicable.

## Supplementary Materials

The following supporting information can be downloaded at: https://www.mdpi.com/article/10.3390/membranes13030337/s1, Figure S1: Average thickness of synthesized MgO nanosheet estimated from FESEM images.; Figure S2: (a) Magnified spectra from 600 to 1700 cm^−1^ (b) difference in the intensity of the peak in the region from 600 to 1700 cm^−1^; Table S1: Thickness of the membranes fabricated by solvent evaporation method.

## References

[1] (2021). IEA Global Energy Review. https://www.iea.org/reports/global-energy-review-2021.

[2] Le Quéré C., Peters G.P., Friedlingstein P., Andrew R.M., Canadell J.G., Davis S.J., Jackson R.B., Jones M.W. (2021). Fossil CO_2_ Emissions in the Post-COVID-19 Era. Nat. Clim. Chang..

[3] Mustafa J., Farhan M., Hussain M. (2016). CO_2_ Separation from Flue Gases Using Different Types of Membranes. J. Membr. Sci. Technol..

[4] Dharupaneedi S.P., Nataraj S.K., Nadagouda M., Reddy K.R., Shukla S.S., Aminabhavi T.M. (2019). Membrane-Based Separation of Potential Emerging Pollutants. Sep. Purif. Technol..

[5] Farnam M., Mukhtar H., Shariff A.M. (2014). A Review on Glassy Polymeric Membranes for Gas Separation. Appl. Mech. Mater..

[6] Robeson L.M. (1991). Correlation of Separation Factor versus Permeability for Polymeric Membranes. J. Memb. Sci..

[7] Robeson L.M. (2008). The Upper Bound Revisited. J. Memb. Sci..

[8] Ghanem B.S., Hashem M., Harris K.D.M., Msayib K.J., Xu M., Budd P.M., Chaukura N., Book D., Tedds S., Walton A. (2010). Triptycene-Based Polymers of Intrinsic Microporosity: Organic Materials That Can Be Tailored for Gas Adsorption. Macromolecules.

[9] AlQahtani M.S., Mezghani K. (2018). Thermally Rearranged Polypyrrolone Membranes for High-Pressure Natural Gas Separation Applications. J. Nat. Gas Sci. Eng..

[10] Lee W.H., Seong J.G., Hu X., Lee Y.M. (2020). Recent Progress in Microporous Polymers from Thermally Rearranged Polymers and Polymers of Intrinsic Microporosity for Membrane Gas Separation: Pushing Performance Limits and Revisiting Trade-off Lines. J. Polym. Sci..

[11] Yong W.F., Zhang H. (2021). Recent Advances in Polymer Blend Membranes for Gas Separation and Pervaporation. Prog. Mater. Sci..

[12] Jusoh N., Yeong Y.F., Chew T.L., Lau K.K., Shariff A.M. (2016). Current Development and Challenges of Mixed Matrix Membranes for CO_2_/CH_4_ Separation. Sep. Purif. Rev..

[13] Liu M., Nothling M.D., Zhang S., Fu Q., Qiao G.G. (2022). Thin Film Composite Membranes for Postcombustion Carbon Capture: Polymers and Beyond. Prog. Polym. Sci..

[14] Tantekin-Ersolmaz Ş.B., Atalay-Oral Ç., Tatlier M., Erdem-Şenatalar A., Schoeman B., Sterte J. (2000). Effect of Zeolite Particle Size on the Performance of Polymer-Zeolite Mixed Matrix Membranes. J. Memb. Sci..

[15] Madaeni S.S., Badieh M.M.S., Vatanpour V., Ghaemi N. (2012). Effect of Titanium Dioxide Nanoparticles on Polydimethylsiloxane/Polyethersulfone Composite Membranes for Gas Separation. Polym. Eng. Sci..

[16] Nour M., Berean K., Balendhran S., Ou J.Z., Plessis J.D., McSweeney C., Bhaskaran M., Sriram S., Kalantar-zadeh K. (2013). CNT/PDMS Composite Membranes for H_2_ and CH_4_ Gas Separation. Int. J. Hydrog. Energy.

[17] Wang H., Ni Y., Dong Z., Zhao Q. (2021). A Mechanically Enhanced Metal-Organic Framework/PDMS Membrane for CO_2_/N_2_ Separation. React. Funct. Polym..

[18] Feijani E.A., Tavassoli A., Mahdavi H., Molavi H. (2018). Effective Gas Separation through Graphene Oxide Containing Mixed Matrix Membranes. J. Appl. Polym. Sci..

[19] Wu H., Zamanian M., Kruczek B., Thibault J. (2020). Gas Permeation Model of Mixed-Matrix Membranes with Embedded Impermeable Cuboid Nanoparticles. Membranes.

[20] Zainuddin M.I.F., Ahmad A.L. (2022). Mixed Matrix Membrane Development Progress and Prospect of Using 2D Nanosheet Filler for CO_2_ Separation and Capture. J. CO2 Util..

[21] Zahri K., Wong K.C., Goh P.S., Ismail A.F. (2016). Graphene Oxide/Polysulfone Hollow Fiber Mixed Matrix Membranes for Gas Separation. RSC Adv..

[22] Yang E., Goh K., Chuah C.Y., Wang R., Bae T.H. (2020). Asymmetric Mixed-Matrix Membranes Incorporated with Nitrogen-Doped Graphene Nanosheets for Highly Selective Gas Separation. J. Memb. Sci..

[23] Mohammed S.A., Nasir A.M., Aziz F., Kumar G., Sallehhudin W., Jaafar J., Lau W.J., Yusof N., Salleh W.N.W., Ismail A.F. (2019). CO_2_/N_2_ Selectivity Enhancement of PEBAX MH 1657/Aminated Partially Reduced Graphene Oxide Mixed Matrix Composite Membrane. Sep. Purif. Technol..

[24] Asim M., Khan A., Helal A., Alshitari W., Akbar U.A., Khan M.Y. (2021). A 2D Graphitic-Polytriaminopyrimidine (G-PTAP)/Poly(Ether-block-amide) Mixed Matrix Membrane for CO_2_ Separation. Chem. An. Asian J..

[25] Liu M., Gurr P.A., Fu Q., Webley P.A., Qiao G.G. (2018). Two-Dimensional Nanosheet-Based Gas Separation Membranes. J. Mater. Chem. A.

[26] Kamble A.R., Patel C.M., Murthy Z.V.P. (2021). A Review on the Recent Advances in Mixed Matrix Membranes for Gas Separation Processes. Renew. Sustain. Energy Rev..

[27] Gou Y., Xiao L., Yang Y., Guo X., Zhang F., Zhu W., Xiao Q. (2021). Incorporation of Open-Pore MFI Zeolite Nanosheets in Polydimethylsiloxane (PDMS) to Isomer-Selective Mixed Matrix Membranes. Microporous Mesoporous Mater..

[28] Shen G., Zhao J., Guan K., Shen J., Jin W. (2017). Highly Efficient Recovery of Propane by Mixed-Matrix Membrane via Embedding Functionalized Graphene Oxide Nanosheets into Polydimethylsiloxane. AIChE J..

[29] Jafari A., Mortaheb H.R., Gallucci F. (2022). Performance of Octadecylamine-Functionalized Graphene Oxide Nanosheets in Polydimethylsiloxane Mixed Matrix Membranes for Removal of Toluene from Water by Pervaporation. J. Water Process Eng..

[30] Pacchioni G. (1993). Physisorbed and Chemisorbed CO_2_ at Surface and Step Sites of the MgO(100) Surface. Surf. Sci..

[31] Wan Isahak W.N.R., Ramli Z.A.C., Mohamed Hisham M.W., Yarmo M.A. (2013). Magnesium Oxide Nanoparticles on Green Activated Carbon as Efficient CO_2_ Adsorbent. AIP Conf. Proc..

[32] Ruhaimi A.H., Aziz M.A.A., Jalil A.A. (2021). Magnesium Oxide-Based Adsorbents for Carbon Dioxide Capture: Current Progress and Future Opportunities. J. CO2 Util..

[33] Hosseini S.S., Li Y., Chung T.S., Liu Y. (2007). Enhanced Gas Separation Performance of Nanocomposite Membranes Using MgO Nanoparticles. J. Memb. Sci..

[34] Momeni S.M., Pakizeh M. (2013). Preparation, Characterization and Gas Permeation Study of PSf/MgO Nanocomposite Membrane. Braz. J. Chem. Eng..

[35] Li P., Chen R., Lin Y., Li W. (2021). General Approach to Facile Synthesis of MgO-Based Porous Ultrathin Nanosheets Enabling High-Efficiency CO_2_ Capture. Chem. Eng. J..

[36] Heo Y.J., Park S.J. (2017). Facile Synthesis of MgO-Modified Carbon Adsorbents with Microwave-Assisted Methods: Effect of MgO Particles and Porosities on CO_2_ Capture. Sci. Rep..

[37] Choe J.H., Kim H., Hong C.S. (2021). MOF-74 Type Variants for CO_2_ capture. Mater. Chem. Front..

[38] Roh E., Subiyanto I., Choi W., Park Y.C., Cho C.H., Kim H. (2021). CO_2_/N_2_ and O_2_/N_2_ Separation Using Mixed-Matrix Membranes with MOF-74 Nanocrystals Synthesized Via Microwave Reactions. Bull. Korean Chem. Soc..

[39] Ahmad A.L., Otitoju T.A., Ooi B.S. (2019). Hollow Fiber (HF) Membrane Fabrication: A Review on the Effects of Solution Spinning Conditions on Morphology and Performance. J. Ind. Eng. Chem..

[40] Choi S.-H., Tasselli F., Jansen J.C., Barbieri G., Drioli E. (2010). Effect of the Preparation Conditions on the Formation of Asymmetric Poly(Vinylidene Fluoride) Hollow Fibre Membranes with a Dense Skin. Eur. Polym. J..

[41] Ismail A.F., Dunkin I.R., Gallivan S.L., Shilton S.J. (1999). Production of Super Selective Polysulfone Hollow Fiber Membranes for Gas Separation. Polymer.

[42] Robeson L.M., Liu Q., Freeman B.D., Paul D.R. (2015). Comparison of Transport Properties of Rubbery and Glassy Polymers and the Relevance to the Upper Bound Relationship. J. Memb. Sci..

[43] Scholes C.A., Stevens G.W., Kentish S.E. (2012). Membrane Gas Separation Applications in Natural Gas Processing. Fuel.

[44] Zainuddin M.I.F., Ahmad A.L. (2022). Impact of Dope Extrusion Rate and Multilayer Polydimethylsiloxane Coating on Asymmetric Polyethersulfone Hollow Fiber Membrane for CO_2_/N_2_ and CO_2_/CH_4_ Separation. Asia-Pacific J. Chem. Eng..

[45] Kotal M., Bhowmick A.K. (2015). Polymer Nanocomposites from Modified Clays: Recent Advances and Challenges. Prog. Polym. Sci..

[46] Alavi S.A., Kargari A., Sanaeepur H., Karimi M. (2017). Preparation and Characterization of PDMS/Zeolite 4A/PAN Mixed Matrix Thin Film Composite Membrane for CO_2_/N_2_ and CO_2_/CH_4_ Separations. Res. Chem. Intermed..

[47] Henis J.M.S., Tripodi M.K. (1981). Composite Hollow Fiber Membranes for Gas Separation: The Resistance Model Approach. J. Memb. Sci..

[48] Da Silva E.A., Windmöller D., Silva G.G., De Souza Figueiredo K.C. (2017). Polydimethylsiloxane Membranes Containing Multi-Walled Carbon Nanotubes for Gas Separation. Mater. Res..

[49] Qureshi M., Chetia T.R., Ansari M.S., Soni S.S. (2015). Enhanced Photovoltaic Performance of Meso-Porous SnO_2_ Based Solar Cells Utilizing 2D MgO Nanosheets Sensitized by a Metal-Free Carbazole Derivative. J. Mater. Chem. A.

[50] Park C.H., Lee J.H., Jung J.P., Kim J.H. (2017). Mixed Matrix Membranes Based on Dual-Functional MgO Nanosheets for Olefin/Paraffin Separation. J. Memb. Sci..

[51] Jin S., Bang G., Lee C.H. (2020). Unusual Morphology Transformation and Basicity of Magnesium Oxide Controlled by Ageing Conditions and Its Carbon Dioxide Adsorption. J. CO2 Util..

[52] Ruben B., Elisa M., Leandro L., Victor M., Gloria G., Marina S., Mian S.K., Pandiyan R., Nadhira L. (2017). Oxygen Plasma Treatments of Polydimethylsiloxane Surfaces: Effect of the Atomic Oxygen on Capillary Flow in the Microchannels. Micro Nano Lett..

[53] Alzahid Y.A., Mostaghimi P., Gerami A., Singh A., Privat K., Amirian T., Armstrong R.T. (2018). Functionalisation of Polydimethylsiloxane (PDMS)- Microfluidic Devices Coated with Rock Minerals. Sci. Rep..

[54] Adhikari N.M., Tuladhar A., Wang Z., De Yoreo J.J., Rosso K.M. (2021). No Hydrogen Bonding between Water and Hydrophilic Single Crystal MgO Surfaces?. J. Phys. Chem. C.

[55] Firpo G., Angeli E., Repetto L., Valbusa U. (2015). Permeability Thickness Dependence of Polydimethylsiloxane (PDMS) Membranes. J. Memb. Sci..

[56] Firpo G., Angeli E., Guida P., Savio R.L., Repetto L., Valbusa U. (2018). Gas Permeation through Rubbery Polymer Nano-Corrugated Membranes. Sci. Rep..

[57] Deepa B., Rajendran V. (2018). Investigation of Organic Solvents Assisted Nano Magnesium Oxide Nanoparticles and Their Structural, Morphological, Optical and Antimicrobial Performance. Mater. Res. Express.

[58] Tong Z., Li L., Li Y., Wang Q., Cheng X. (2021). The Effect of in Situ Synthesis of MgO Nanoparticles on the Thermal Properties of Ternary Nitrate. Materials.

[59] Jusoh N., Yeong Y.F., Lau K.K., Shariff A.M. (2017). Enhanced Gas Separation Performance Using Mixed Matrix Membranes Containing Zeolite T and 6FDA-Durene Polyimide. J. Memb. Sci..

[60] Chang Y.W., Chang B.K. (2018). Influence of Casting Solvents on Sedimentation and Performance in Metal–Organic Framework Mixed-Matrix Membranes. J. Taiwan Inst. Chem. Eng..

[61] Shamsabadi A.A., Kargari A., Babaheidari M.B., Laki S., Ajami H. (2013). Role of Critical Concentration of PEI in NMP Solutions on Gas Permeation Characteristics of PEI Gas Separation Membranes. J. Ind. Eng. Chem..

[62] Idris A., Man Z., Maulud A., Khan M. (2017). Effects of Phase Separation Behavior on Morphology and Performance of Polycarbonate Membranes. Membranes.

[63] Johnson L.M., Gao L., Shields C.W., Smith M., Efimenko K., Cushing K., Genzer J., López G.P. (2013). Elastomeric Microparticles for Acoustic Mediated Bioseparations. J. Nanobiotechnology.

[64] Balakrishnan G., Velavan R., Mujasam Batoo K., Raslan E.H. (2020). Microstructure, Optical and Photocatalytic Properties of MgO Nanoparticles. Results Phys..

[65] El-Sayyad G.S., Mosallam F.M., El-Batal A.I. (2018). One-Pot Green Synthesis of Magnesium Oxide Nanoparticles Using Penicillium Chrysogenum Melanin Pigment and Gamma Rays with Antimicrobial Activity against Multidrug-Resistant Microbes. Adv. Powder Technol..

[66] Wong C.W., Chan Y.S., Jeevanandam J., Pal K., Bechelany M., Abd Elkodous M., El-Sayyad G.S. (2020). Response Surface Methodology Optimization of Mono-Dispersed MgO Nanoparticles Fabricated by Ultrasonic-Assisted Sol–Gel Method for Outstanding Antimicrobial and Antibiofilm Activities. J. Clust. Sci..

[67] Zahir M.H., Rahman M.M., Irshad K., Rahman M.M. (2019). Shape-Stabilized Phase Change Materials for Solar Energy Storage: MgO and Mg(OH)_2_ Mixed with Polyethylene Glycol. Nanomaterials.

[68] Glisenti A., Frasson A., Galenda A., Ferroni M., Concina I., Natilea M.M. (2010). Synthesis and Characterization of Ag/CeO_2_ Nanocomposites. Mater. Res. Soc. Symp. Proc..

[69] Wetteland C.L., de Jesus Sanchez J., Silken C.A., Nguyen N.Y.T., Mahmood O., Liu H. (2018). Dissociation of Magnesium Oxide and Magnesium Hydroxide Nanoparticles in Physiologically Relevant Fluids. J. Nanoparticle Res..

[70] Gulková D., Šolcová O., Zdražil M. (2004). Preparation of MgO Catalytic Support in Shaped Mesoporous High Surface Area Form. Microporous Mesoporous Mater..

[71] Al-Harbi L.M., Darwish M.S.A., Khowdiary M.M., Stibor I. (2018). Controlled Preparation of Thermally Stable Fe-Poly(Dimethylsiloxane) Composite by Magnetic Induction Heating. Polymers.

[72] Haider B., Dilshad M.R., Akram M.S., Islam A., Kaspereit M. (2021). Novel Polydimethylsiloxane Membranes Impregnated with SAPO-34 Zeolite Particles for Gas Separation. Chem. Pap..

[73] Suleman M.S., Lau K.K., Yeong Y.F. (2016). Characterization and Performance Evaluation of PDMS/PSF Membrane for CO_2_/CH_4_ Separation under the Effect of Swelling. Procedia Eng..

[74] Zalewski K., Chyłek Z., Trzciński W.A. (2021). A Review of Polysiloxanes in Terms of Their Application in Explosives. Polymers.

[75] Selyanchyn R., Ariyoshi M., Fujikawa S. (2018). Thickness Effect on CO_2_/N_2_ Separation in Double Layer Pebax-1657^®^/PDMS Membranes. Membranes.

[76] Mohr J.M., Paul D.R. (1991). Effect of Casting Solvent on the Permeability of Poly(4-Methyl-1-Pentene). Polymer.

[77] Kulak H., Thür R., Vankelecom I.F.J. (2022). MOF/Polymer Mixed-Matrix Membranes Preparation: Effect of Main Synthesis Parameters on CO_2_/CH_4_ Separation Performance. Membranes.

[78] Chen B., Wan C., Kang X., Chen M., Zhang C., Bai Y., Dong L. (2019). Enhanced CO_2_ Separation of Mixed Matrix Membranes with ZIF-8@GO Composites as Fillers: Effect of Reaction Time of ZIF-8@GO. Sep. Purif. Technol..

[79] Aroon M.A., Ismail A.F., Matsuura T., Montazer-Rahmati M.M. (2010). Performance Studies of Mixed Matrix Membranes for Gas Separation: A Review. Sep. Purif. Technol..

[80] Zornoza B., Téllez C., Coronas J. (2011). Mixed Matrix Membranes Comprising Glassy Polymers and Dispersed Mesoporous Silica Spheres for Gas Separation. J. Memb. Sci..

[81] DeRocher J.P., Gettelfinger B.T., Wang J., Nuxoll E.E., Cussler E.L. (2005). Barrier Membranes with Different Sizes of Aligned Flakes. J. Memb. Sci..

[82] Kang Z., Peng Y., Hu Z., Qian Y., Chi C., Yeo L.Y., Tee L., Zhao D. (2015). Mixed Matrix Membranes Composed of Two-Dimensional Metal–Organic Framework Nanosheets for Pre-Combustion CO_2_ Capture: A Relationship Study of Filler Morphology versus Membrane Performance. J. Mater. Chem. A.

[83] Ehsani A., Pakizeh M. (2016). Synthesis, Characterization and Gas Permeation Study of ZIF-11/Pebax® 2533 Mixed Matrix Membranes. J. Taiwan Inst. Chem. Eng..

[84] Li T., Pan Y., Peinemann K.V., Lai Z. (2013). Carbon Dioxide Selective Mixed Matrix Composite Membrane Containing ZIF-7 Nano-Fillers. J. Memb. Sci..

[85] Gunasakaran A., Jafa J., Saalah S., Sipaut C.S., Yusof N., Aziz F., Ismail A.F., Bilad M.R., Yahya N.Y., Ismail N.M. Activated Carbon and Halloysite Nanotubes Membrane for CO_2_ and CH_4_ Separation. Proceedings of the IOP Conference Series: Materials Science and Engineering.

[86] Molki B., Aframehr W.M., Bagheri R., Salimi J. (2018). Mixed Matrix Membranes of Polyurethane with Nickel Oxide Nanoparticles for CO_2_ Gas Separation. J. Memb. Sci..

[87] Usman M., Khan M.Y., Anjum T., Khan A.L., Hoque B., Helal A., Hakeem A.S., Al-Maythalony B.A. (2022). Controlled Covalent Functionalization of ZIF-90 for Selective CO_2_ Capture & Separation. Membranes.

[88] Houben M., Kloos J., van Essen M., Nijmeijer K., Borneman Z. (2022). Systematic Investigation of Methods to Suppress Membrane Plasticization during CO_2_ Permeation at Supercritical Conditions. J. Memb. Sci..

[89] Askari M. (2017). CO_2_/CH_4_ Sorption Behavior of Glassy Polymeric Membranes Based on Dual Mode Sorption Model. Bull. Société R. Sci. Liège.

[90] Jamil A., Zulfiqar M., Arshad U., Mahmood S., Iqbal T., Rafiq S., Iqbal M.Z. (2020). Development and Performance Evaluation of Cellulose Acetate-Bentonite Mixed Matrix Membranes for CO_2_ Separation. Adv. Polym. Technol..

[91] Wang M., Wang Z., Li N., Liao J., Zhao S., Wang J., Wang S. (2015). Relationship between Polymer-Filler Interfaces in Separation Layers and Gas Transport Properties of Mixed Matrix Composite Membranes. J. Memb. Sci..

[92] Wang D., Ying Y., Zheng Y., Pu Y., Yang Z., Zhao D. (2022). Induced Polymer Crystallinity in Mixed Matrix Membranes by Metal-Organic Framework Nanosheets for Gas Separation. J. Membr. Sci. Lett..

[93] Dilshad M.R., Islam A., Haider B., Sabir A., Ijaz A., Khan R.U., Durrani A.K. (2020). Novel PVA/PEG Nano-Composite Membranes Tethered with Surface Engineered Multi-Walled Carbon Nanotubes for Carbon Dioxide Separation. Microporous Mesoporous Mater..

[94] Dilshad M.R., Islam A., Haider B., Sajid M., Ijaz A., Khan R.U., Khan W.G. (2021). Effect of Silica Nanoparticles on Carbon Dioxide Separation Performances of PVA/PEG Cross-Linked Membranes. Chem. Pap..

[95] Hussain M., König A. (2012). Mixed-Matrix Membrane for Gas Separation: Polydimethylsiloxane Filled with Zeolite. Chem. Eng. Technol..

[96] Rezakazemi M., Shahidi K., Mohammadi T. (2012). Hydrogen Separation and Purification Using Crosslinkable PDMS/Zeolite A Nanoparticles Mixed Matrix Membranes. Int. J. Hydrog. Energy.

