# Supplementary material for: Polydimethylsiloxane/Magnesium Oxide Nanosheet Mixed Matrix Membrane for CO2 Separation Application
Source: Membranes (Basel). 2023 Mar 14;13(3):337. doi: 10.3390/membranes13030337 (PMC10051079; doi:10.3390/membranes13030337)
Supplement: Supplementary file 1 [file membranes-13-00337-s001.zip › membranes-2224461-supplementary.pdf]

membranes

Article

Polydimethylsiloxane/Magnesium Oxide Nanosheet Mixed Matrix Membrane for CO_2 Separation Application

Muhd Izzudin Fikry Zainuddin [1], Abdul Latif Ahmad [1,*] and Meor Muhammad Hafiz Shah Buddin [2]

[1] School of Chemical Engineering, Universiti Sains Malaysia Engineering Campus, Nibong Tebal 14300, Pulau Pinang, Malaysia
[2] School of Chemical Engineering, College of Engineering, Universiti Teknologi MARA, Shah Alam 40450, Selangor, Malaysia
* Correspondence: chlatif@usm.my

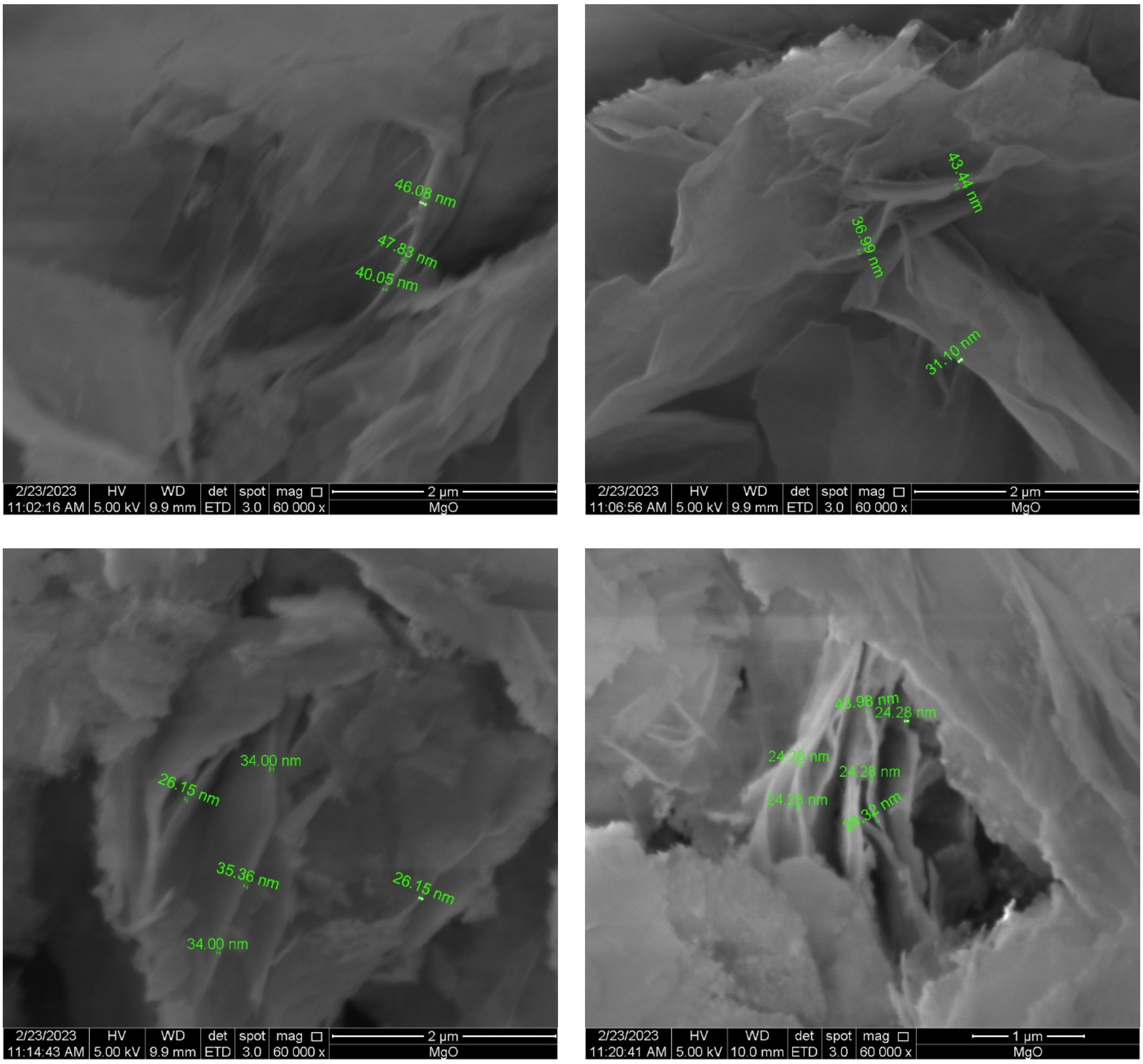

Figure S1. Average thickness of synthesized MgO nanosheet estimated from FESEM images.

Table S1. Thickness of the membranes fabricated by solvent evaporation method.

Membrane	Thickness (μm)
M1	424 ± 30
M2	262 ± 2
M3	256 ± 1
M4	311 ± 6
M5	300 ± 16
M6	441 ± 1

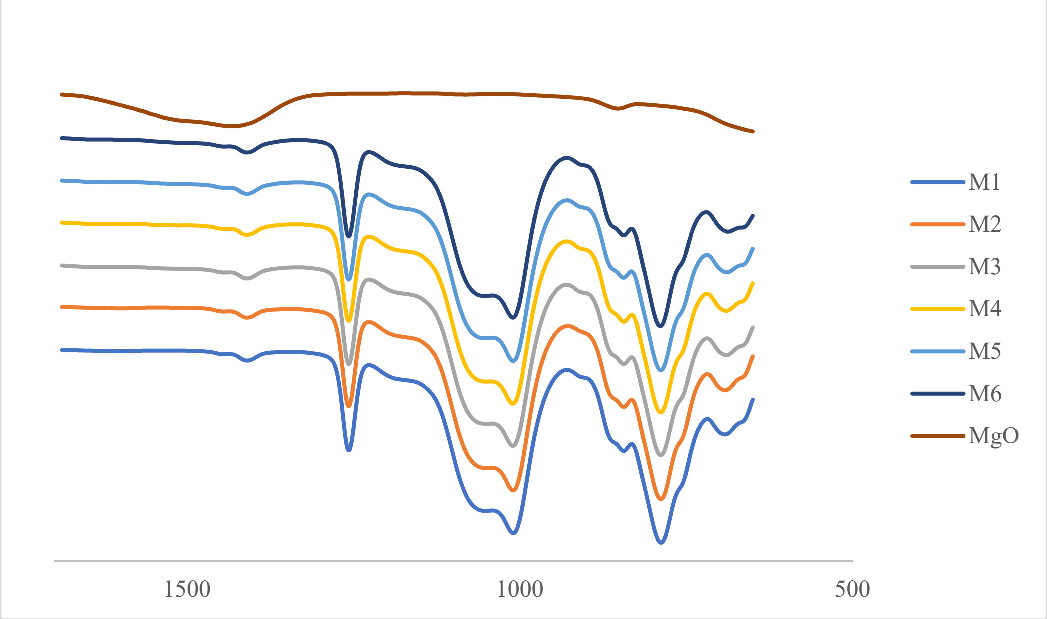

(a)

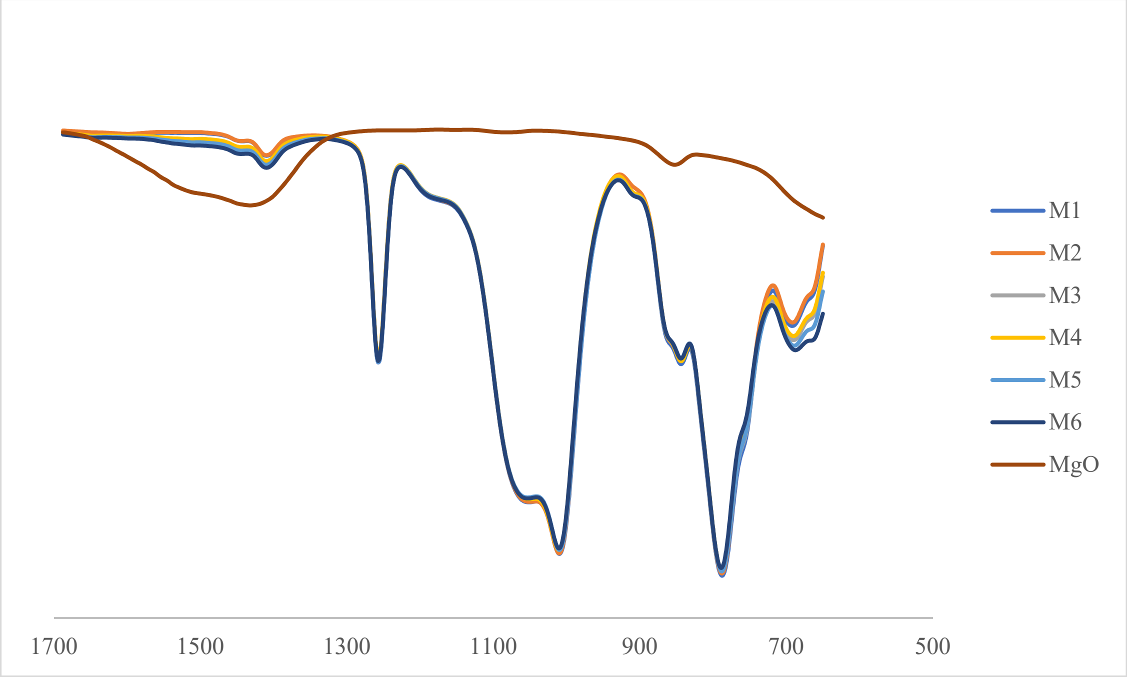

(b)

Figure S2. (**a**) Magnified spectra from 600 to 1700 cm^{-1} (**b**) difference in the intensity of the peak in the region from 600 to 1700 cm^{-1}.
